# Enteric-coated insulin microparticles delivered by lipopeptides of iturin and surfactin

**DOI:** 10.1080/10717544.2017.1413443

**Published:** 2017-12-10

**Authors:** Xiaoying Xing, Xiuyun Zhao, Jia Ding, Dongming Liu, Gaofu Qi

**Affiliations:** ^a^ College of Life Science and Technology, Huazhong Agricultural University Wuhan China; ^b^ College of Veterinary Medicine, Huazhong Agricultural University Wuhan China; ^c^ Biomedical Center, Huazhong Agricultural University Wuhan China

**Keywords:** Oral insulin, microparticles, iturin, surfactin, acryl-eze

## Abstract

Surfactin, a lipopeptide produced by *Bacillus* species, has been used for the oral delivery of insulin. In this study, another lipopeptide of iturin was tested for its ability to orally delivery insulin alone or plus surfactin. Iturin could form co-precipitate with insulin at acidic pH values. After treatment by ultrasonification, the structure of coprecipitate was destroyed that led to a significant decrease in hypoglycemic effect after oral administration. Iturin weakly binds to (*Kd* = 257 μM) and induce insulin structure more compact that is favorable for insulin uptake by the intestine. After being coated with Acryl-Eze by lyophilization, the coprecipitate formed the spherical enteric-coated insulin microparticles delivered by iturin with a relative oral bioavailability of 6.84% in diabetic mice. For further improving oral hypoglycemic effect, surfactin was added to form the spherical enteric-coated insulin microparticles in a formulation containing insulin, Acryl-Eze, iturin and surfactin at a ratio of 1:1:0.5: 0.5 (w/w), with an insulin encapsulation efficiency of 66.22%. The enteric-coated insulin microparticles delivered by iturin plus surfactin showed a classical profile for controlled release in the intestine with a relative bioavailability of 7.67% after oral administration, which could effectively control the postprandial blood glucose at a level about 50% of the initial one just like the subcutaneous injection. Collectively, iturin plus surfactin is more efficient for oral delivering insulin than the sole one, and the resultant enteric-coated insulin microparticles are potential for the development of oral insulin to control postprandial blood glucose in diabetic patients.

## Introduction

Diabetes mellitus is one of the most common chronic metabolic disease due to lack of insulin (INS) (T1DM) or INS resistance (T2DM) (Sousa et al., 2015; Wong et al., 2016). Diabetes can be controlled by subcutaneous (*s.c.*) injection with INS (Lopes et al., 2015; Akbari et al., 2016). Generally, the typical diabetic patients require more than 60,000 injections throughout lives, but the multiple daily injection is with low compliance for patients, such as painful, inconvenient and uncomfortable (Wong et al., 2016). Furthermore, this route has several side effects such as peripheral hyperinsulinemia, hypoglycemia, weight gain, fat deposition, etc. (Lim et al., 2014; Kanzarkar et al., 2015; Sousa et al., 2015). For these reasons, numerous researchers attempted to develop noninvasive routes for delivering INS (Nur & Vasiljevic, 2017).

Oral route, one of the noninvasive routes for INS delivery, has the highest patient compliance by mimicking the normal physiological route of INS secretion to avoid peripheral hyperinsulinemia and other adverse effects (Ismail & Csóka, 2017; Nur & Vasiljevic, 2017). Upon oral administration, INS is directly absorbed from the intestinal lumen to the liver via the portal circulation, mimicking the effect of endogenous INS in a glucose liver metabolism (Lim et al., 2014; Kanzarkar et al., 2015; Sousa et al., 2015). Accordingly, oral delivery is considered an ideal route for INS administration (Lopes et al., 2015), but along the gastrointestinal (GI) tract, there are several barriers to be overtaken such as INS digestion and absorption obstacle (Zijlstra et al., 2014; Fonte et al., 2015; Sharma et al., 2015; Sousa et al., 2015; Wong et al., 2016; Nur & Vasiljevic, 2017).

To overcome barriers from the GI tract, several strategies have been developed including the employment of protease inhibitors, permeation enhancers, mucoadhesive polymers, particulate carrier delivery systems or even a combination of the aforementioned strategies (Wong et al., 2016). Even great advances have been achieved, the intestinal epithelium permeability is still the main obstacle to overcome for the development of oral INS (Fonte et al., 2015; Lopes et al., 2015).

Previously, we used the *Bacillus*-produced lipopeptide of surfactin (SFN) to improve INS uptake by the intestine and found the natural SFN and chemically synthesized analogs are all favorable for promoting INS uptake by the intestine (Zhang et al., 2016, 2017). Addition to SFN, iturin (ITU) is another lipopeptide produced by *Bacillus* species with a well known anti-fungal activity (Zhao et al., 2017). SFN is a very powerful biosurfactant containing a hydrophilic head group of cyclic heptapeptide that is linked to a beta-hydroxy fatty acid of 13–15 C (Figure S1a, Supplemental File). ITU is also a cyclic lipoheptapeptide that is linked to a fatty acid chain with 14–17 C (Figure S1b, Supplemental File) (Patel et al., 2015). Due to the amphipathic structure, both SFN and ITU can disturb the integrity and permeability of membrane by a detergent-like mechanism (Inès & Dhouha, 2015; Otzen, 2017).

One important strategy to overcome harsh environment of the stomach is to use enteric coating materials such as Acryl-Eze, which is a commercially available acrylic coating system, combining the benefits of a globally accepted enteric acrylic polymer Eudragit^®^ L10055 (Hashmat et al., 2008; Bruce et al., 2010; Shen et al., 2011). Acryl-Eze is generally used for enteric coating chemical drugs that are stable toward high temperature and organic solvents in the progress of coating. However, INS is a protein drug, which is sensitive to these harsh environments. Thereby, the harsh environments should be avoided for enteric coating INS by Acryl-Eze.

In this study, we developed a novel strategy to produce the orally delivered INS microparticles (iMSs) by utilization of lipopeptides (ITU and SFN). After being enteric-coated with Acryl-Eze by lyophilization, the produced enteric-coated iMSs delivered by lipopeptides were very efficient for oral delivery of INS showing a significant effect on the control of postprandial blood glucose by a sustained release profile in T1DM mice.

## Materials and methods

### Materials and reagents

Recombinant human INS (27 IU/mg) was a gift from JS Bioway pharmaceutical Co., Ltd (Changzhou City, China). Streptozotocin and Sephadex G25 was purchased from Sigma-Aldrich (USA). Human INS enzyme-linked immunosorbent assay (ELISA) Kits were purchased from RayBiotech (Norcross, USA). SFN variant containing a fatty acid chain with 14 carbons was chemically synthesized by Chinese Peptide Company (Hangzhou, China) for a purify >90% (Zhang et al., 2017). Acryl-Eze was a gift from Prof. Hong Liu in Hubei University, Wuhan, China. Monolith NT-647 labeling Kits were purchased from NanoTemper Technologies, Germany. Other chemicals were of analytical grade supplied by Sinopharm Chemical Reagent (China).

### Fermentation of *Bacillus amyloliquefaciens* for production of iturin (ITU)

For purification of ITU, *B. amyloliquefaciens* WH1 was cultured in 200 ml of medium consisting of (per liter) 12 g soybean powder, 2 g NH_4_NO_3_, 2 g Na_2_HPO_4_, 15 g corn powder at pH 7.0 in a 1 L-flask at 28 °C and 200 rpm for 48 h. Thereafter, the supernatant of culture was adjusted to pH 2.0 for precipitation, then the precipitate was dissolved in distilled water and extracted by the same volume of n-butanol. The extracted substance was loaded into Sephadex G-25 column then eluted by distilled water. The eluted fractions with ITU were monitored by detecting their anti-fungal activity against *Fusarium oxysporum*. The fraction with strong anti-fungal activity was loaded into silica gel (FCP-200) column then eluted by *n*-butanol: ethanol: acetic acid: water = 30:70:5:20 (v/v), and the elution containing ITU was monitored by detecting the anti-fungal activity as above. The fraction with antifungal activity was analyzed by HPLC on an Agilent ZORBAX Eclipse Plus C18 column (4.6 × 250 mm, 5 μm), using the mobile phase 20% acetonitrile/0.1% trifluoroacetic acid. Finally, the purified ITU was identified by MALDI-TOF-MS (Li et al., 2006).

### Induction of diabetic mice

Balb/C mice (5–6 weeks old, 18–20 g body weight) were purchased from the center for disease prevention and control of Hubei province (Wuhan, China) following the University Ethics Committee’s guidelines. The mice were intraperitoneally injected with streptozotocin (70 mg/kg) for induction of T1DM (Zhang et al., 2016).

### Enteric-coated INS microparticles delivered by ITU(iMSs-ITU)

Previously, we found INS can form coprecipitate with SFN in acidic conditions (Zhang et al., 2016). Here, 1 mg INS was also mixed with 1 mg ITU in 1 ml of 6% (w/v) mannitol solution (pH 3.5) to form the coprecipitate of INS and ITU. Thereafter, the mixture was freeze-dried to iMSs-ITU, then resuspended in 1 ml Acryl-Eze (1 mg/ml, pH 3.5), following by lyophilization again to form the enteric-coated iMSs-ITU.

To know whether INS activity was influenced by Acryl-Eze, the enteric-coated iMSs-ITU was dissolved in phosphate buffer saline (PBS, pH 7.0) for subcutaneous (*s.c.*) injection of diabetic mice (1 IU/kg body weight, 7 mice per group). Another group was injected with the same dose of freshly prepared INS as control. Blood was collected from the tail then detect by an ACCU-CHEK Active glucometer (Roche) prior to injection as the fasting glucose level, and at different time intervals post injection. Results are presented as the % reduction from the initial value by Equation (1):

(1) $$\%\text{ change}\;=\;\left[\left(F-P_{t}\right)/F\right]\;\times \;\text{1}00$$

where, *F* is the fasting blood glucose level, *P_t_* is the blood glucose level at time (t) after injection.

The iMSs-ITU with or without coating by Acryl-Eze was also suspended in water for oral administration of diabetic mice (7 mice per group) at an INS dose of 45 IU/kg by an oral feeding needle. The diabetic mice orally treated with water were used as a control. Thereafter, the blood glucose level was detected for calculating the oral hypoglycemic effect according to Equation (1).

Amounts of Acryl-Eze might influence the oral hypoglycemic effect of enteric-coated iMSs-ITU by determining the thickness of enteric-coating layer. Thereby, the amount of Acryl-Eze for production of enteric-coated iMSs-ITU was determined in this study. One milligram INS was mixed with 1 mg ITU in 1 ml of 6% (w/v) mannitol solution (pH 3.5) to form the co-precipitate of INS and ITU. Thereafter, the mixture was freeze-dried to iMSs-ITU following coated with different amounts of Acryl-Eze (INS:Acryl-Eze =1:0.1, 1:0.5, 1:1, 1:5 and 1:25, w/w, respectively) by lyophilization again. The enteric-coated iMSs-ITU with different amounts of Acryl-Eze were used for oral administration of diabetic mice (7 mice per group) at an INS dose of 45 IU/kg, then the blood glucose level was detected for calculating the oral hypoglycemic effect of enteric-coated iMSs-ITU with different amounts of Acryl-Eze according to Equation (1).

### Interactions between INS and ITU

MST was used to measure the affinity between INS and ITU. INS was labeled by NT-647 red fluorescent dye with Monolith NT-647 labeling Kits. Sixteen dilutions of ITU ranging from 250 μM to 10 mM were titrated against a constant amount of labeled INS (2 μM) in PBS containing 0.05% of Tween 20 (v/v). MST was performed on a Monolith NT.115 instrument (NanoTemper Technologies, Germany), and *Kd* (dissociation constant) value was calculated by Nano Temper software.

The structure of INS influenced by ITU was probed by Jasco J-810 circular dichroism (CD) spectropolarimeter (Jasco, Japan) at 25 °C as described previously (Zhang et al., 2016). Briefly, the mixture of INS and SFN (Table S1, Supplemental File) at pH 7.0 or firstly at pH 3.5 for forming co-precipitate then to pH 7.0 for redissolving, were detected by a CD spectropolarimeter respectively. The final CD spectra were obtained by subtracting sample spectra from the related concentrations of ITU in solution, then converted to molar ellipticity.

The coprecipitate of INS and ITU was also treated by ultrasonification with an ultrasonic instrument (Illumina, USA) for 0, 5, 10, 20 and 40 min on ice, then freeze-dried to iMSs-ITU following coated by Acryl-Eze (INS:Acryl-Eze = 1:1) to from the enteric-coated iMSs-ITU as above. The enteric-coated iMSs-ITU were suspended in water (pH 3.5), then used for determining the particle sizes by Zetasizer Nano-ZS90 (Malvern Instruments, UK) (Sonia & Sharma, 2013), and the oral hypoglycemic effect at an INS dose of 45 IU/kg.

### Enteric-coated iMSs delivered by ITU plus surfactin (iMSs-ITU-SFN)

For higher hypoglycemic effect, SFN was added to orally deliver INS together with ITU to produce the enteric-coated INS microparticles delivered by ITU plus SFN (iMSs-ITU-SFN). 1 mg INS was mixed with 1 mg mixture of ITU plus SFN at different ratios (Table S2, Supplemental File) in 1 ml of 6% (w/v) mannitol solution (pH 3.5) for forming the coprecipitate of INS, ITU and SFN, then the mixture was freeze-dried to powers following coated with Acryl-Eze to produce the enteric-coated iMSs-ITU-SFN as above. The prepared enteric-coated iMSs-ITU-SFN were suspended in water for oral administration of diabetic mice (7 mice per group) at an INS dose of 30 or 45 IU/kg, respectively. The blood glucose level was detected, and the oral hypoglycemic effect was calculated by Equation (1).

### Observation by electron microscopy

INS was mixed with ITU to form the coprecipitate of INS and ITU in mannitol solution, then freeze-dried to iMSs-ITU as above. INS and ITU were also precipitated in mannitol solution at their pI values respectively, then freeze-dried to powers as control. Additionally, the iMSs-ITU-SFN with or without an enteric-coating film of Acryl-Eze were also produced as described above. After negatively staining with sodium phosphotungstate solution (0.2%, w/v), the structural morphology of samples was observed by transmission electron microscopy (TEM) (JEM-1200EX, Japan). The above samples were also mounted on metal stubs using double-sided adhesive tape coated with gold under vacuum, following examined by scanning electron microscopy (SEM) (HITACHI S-2400, Hitachi, Japan) for the surface morphology.

### Release studies of enteric-coated iMSs *in vitro*

Simulated gastric fluid (SGF) and simulated intestinal fluid (SIF) were used for *in vitro* release studies of the enteric-coated iMSs-ITU and iMSs-ITU-SFN, respectively (Mansourpour et al., 2015). Twenty milligram of enteric-coated iMSs-ITU or iMSs-ITU-SFN was suspended in 20 ml of SGF (pH 1.2) or SIF (pH 6.8), then kept in a shaker (50 rpm) at 37 °C. At specified intervals of time, aliquot of sample (200 µl) was withdrawn for observation by a microscope, and the INS content in solution was estimated by ELISA with human INS ELISA Kits. The dissolution medium was replaced with fresh SGF or SIF to maintain the total volume after each withdrawal (Sonia & Sharma, 2013; Li et al., 2014).

### Determining encapsulation efficiency of INS

The enteric-coated iMSs-ITU or iMSs-ITU-SFN containing 1 mg INS was suspended in 5 ml of acidic water (pH 3.5) to dissolve the unencapsulated INS, then the supernatant was collected for determining INS by ELISA as above. The encapsulation efficiency (EE) was calculated according to Equation (2) (García-Díaz et al., 2015)

(2) EE(%) = [(Total amount of INS added - Amount of INS in supernatant)/Total amount of INS added]×100.

### Relative bioavailability of enteric-coated iMSs

Thirty-six diabetic mice were randomly divided into three groups for 12 mice per group. Two groups were orally treated with the enteric-coated iMSs-ITU or iMSs-ITU-SFN at an INS dose of 45 IU/kg, and another group was *s.c.* injected with INS (1 IU/kg) as control. Blood samples were collected prior to administration as the fasting glucose levels, and at different time intervals postadministration. The blood samples were also used for detecting plasma INS concentration by ELISA, then the relative bioavailability (BA) of INS was calculated by Equation (3)

(3) $$\text{BA}\;=\;\left[{\text{AUC}}_{0–\text{8 oral}}\times \;{\left(\text{weight}/\text{dose}\right)}_{\text{oral}}\right]/\left[{\text{AUC}}_{0–\text{8}\;\text{s}.\text{c}.}\times {\left(\text{weight}/\text{dose}\right)}_{\text{s}.\text{c}.}\right]\times \text{1}00\%.$$

AUC_0–8_ is the area under the reduction of blood glucose from 0 to 8 h; weight (kg) is the body weight of mice and dose (IU) is the amount of INS administered to the animals (Zhang et al., 2016).

### Oral glucose tolerance test of enteric-coated iMSs

For oral glucose tolerance test (OGTT), 36 diabetic mice were randomly divided into 3 groups for 12 mice per group. Two groups were orally administered with the enteric-coated iMSs-ITU or iMSs-ITU-SFN at an insulin dose of 45 IU/kg, and another group was orally treated with water as placebo control. One-hour postadministration, all mice were orally given with 200 μl glucose solution (2 g/kg body weight), then the blood glucose level was determined at each time interval.

### Statistical analysis of data

All experiments were repeated triplicates. Data from different groups were compared by a one-way analysis of variance (ANOVA) with significant level of *p < .05, and **p < .01.

## Results

### Purification and characterization of ITU

*B. amyloliquefaciens* WH1 culture was extracted by n-butanol, then the extracted substance was further separated into two fractions (*P*_1_ and *P*_2_) by Sephadex G-25 (Figure S2a, Supplemental File). *P*_1_ showed a strong activity against fungus, which was further separated into 2 parts (*P*_1.1_ and *P*_1.2_) by silica gel column (Figure S2b, Supplemental File). *P*_1.1_ had a strong anti-fungal activity and was identified as iturin (ITU) series with C13, C14 and C15 by HPLC (Figure S2c, Supplemental File) and MALDI-TOF with m/z of 1050.61, 1064.16, 1066.86 and 1080.75 (Figure S2d, Supplemental File), respectively.

### Enteric-coated iMSs-ITU

**Figure 1.**
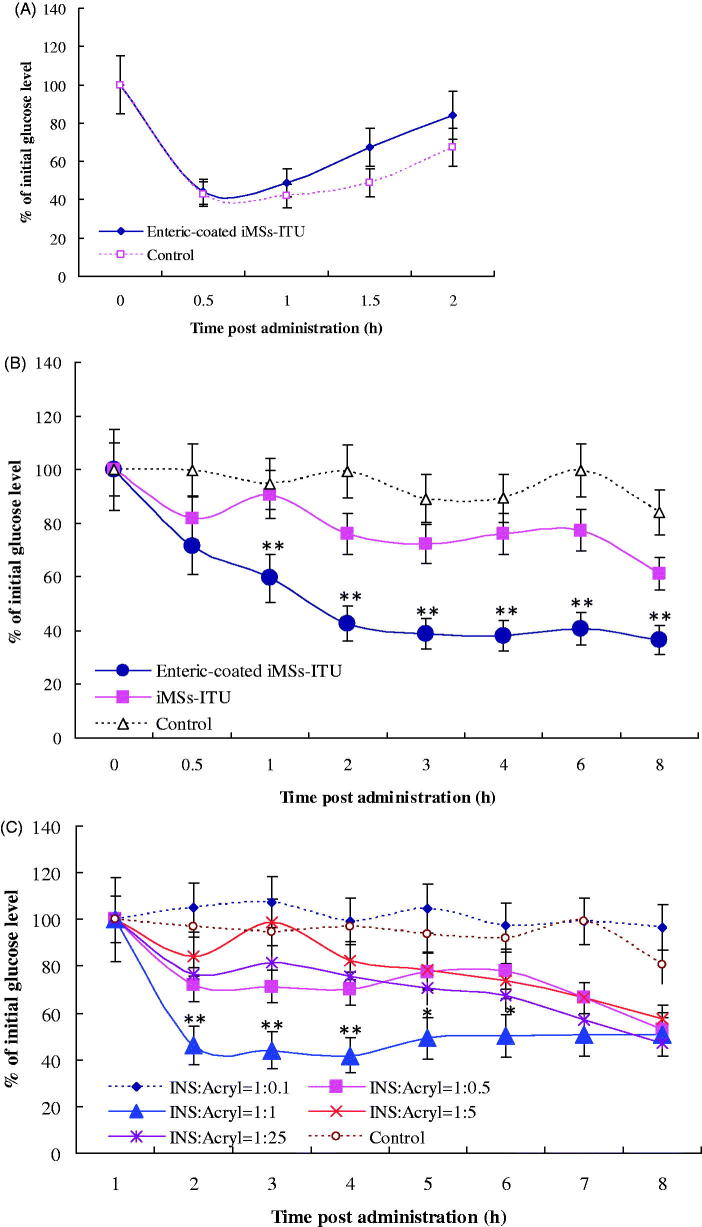
Enteric-coated iMSs-ITU. (A): Hypoglycemic effect of enteric-coated iMSs-ITU for *s.c.* injection. Control: the freshly prepared INS solution. (B): Oral hypoglycemic effect of enteric-coated iMSs-ITU. (C): Oral hypoglycemic effect of iMSs-ITU coated with different amounts of Acryl-Eze. 1:0.1, 1:0.5, 1:1, 1:5 and 1:25 means the ratio of INS to Acryl-Eze (m/m). Reported results are means ± SD of 7 mice. Double stars and single star indicate very significant difference (*p* < .01) and significant difference (*p* < .05) from other groups, respectively.

Just like SFN (Zhang et al., 2016), INS could also form coprecipitate with ITU. Mannitol is a popular additive for lyophilization. Previously, we found that 6% (w/v) mannitol was favorable for lyophilization of coprecipitate (data unpublished). Here, in the presence of mannitol the co-precipitate of INS and ITU was coated with Acryl-Eze to form enteric-coated iMSs-ITU via lyophilization (Figure S3, Supplemental File). *In vivo* assay showed that coating with Acryl-Eze had a slight disturbance on the activity of INS compared to control (Figure 1(A)).

The enteric-coated iMSs-ITU was tested for the oral hypoglycemic effect in diabetic mice. As shown in Figure 1(B), the iMSs-ITU without enteric-coating was found with a slight hypoglycemic effect when compared to control; however, a significant higher hypoglycemic effect was observed for the enteric-coated iMSs-ITU when compared to the one without enteric-coating. One hour post oral administration, the blood glucose level decreased fast to 60.06% of the initial one, then further decreased to 44.83%, 39.89%, 39.89%, 43.07% and 37.88% at 2, 3, 4, 6 and 8 h, respectively. Overall, the enteric-coated iMSs-ITU led to a sustained decrease of blood glucose (about 40% of the initial level) from 2 to 8 h after oral administration. Thereby, enteric-coating with Acryl-Eze was very favorable for improving oral hypoglycemic effect of iMSs-ITU.

The amount of Acryl-Eze was further determined for enteric-coating at an INS: Acryl-Eze ratio of 1:0.1, 1:0.5, 1:1, 1:5 and 1:25 (w/w), respectively. The results showed when the ratio of INS to Acryl-Eze was: (1) 1:0.1, the enteric-coated iMSs-ITU had no obviously hypoglycemic effect compared to control; (2) 1:5 or 1:25, a slightly hypoglycemic effect was observed in the first 4 hours after oral administration; (3) 1:0.5, a strong hypoglycemic effect was found in the first 3 hours post administration; (4) 1:1, the enteric-coated iMSs-ITU showed a very strong hypoglycemic effect after oral administration (Figure 1(C)). The above results indicated that: (1) a small amount of Acryl-Eze (INS:Acryl-Eze = 1:0.1) cannot form an effective enteric coating film for iMSs-ITU; (2) a large amount of Acryl-Eze (INS:Acryl-Eze = 1:5 or 1:25) forms an excessive enteric coating film for iMSs-ITU; and (3) an appropriate amount of Acryl-Eze (INS:Acryl-Eze = 1:1 or 1:0.5) can form a moderate enteric coating film for iMSs-ITU. Accordingly, the ratio of INS to Acryl-Eze was selected as 1:1 (w/w) in the following studies.

### Interactions between INS and ITU

**Figure 2.**
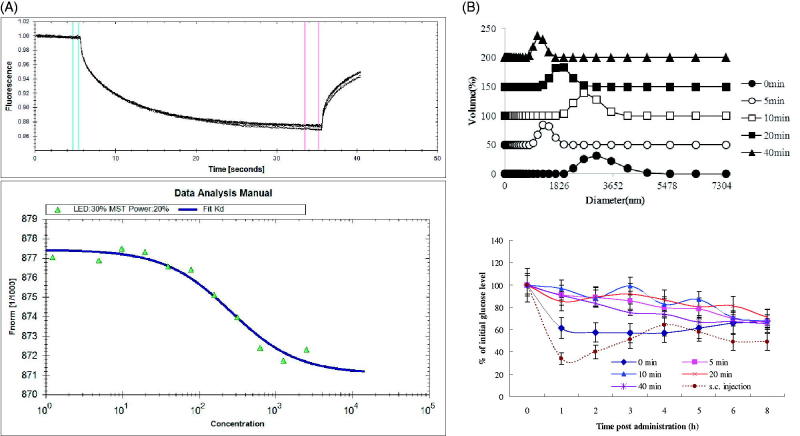
Interactions between insulin (INS) and iturin (ITU). (A): MST analysis of INS-ITU interaction. Binding of INS and ITU at different ratios were studied. Left panel: MST thermogram “thermophoresis + T-jump” settings showing positive (hot to cold region) as well as negative thermophoresis (cold to hot region) for ITU with INS confirming a weak interaction; Right panel: Binding of ITU to fluorescently labeled INS was quantified using Microscale thermophoresis (MST). The horizontal axis is ITU concentration (µM). Sixteen dilutions of ITU ranging from 250 μM to 10 mM were titrated against a constant amount of labeled INS (2 μM). The *Kd* fit graph of INS-ITU interaction demonstrates a steady interaction between INS and ITU with a *Kd* value of 257 μM. (B): Diameter distribution and oral hypoglycemic effect of iMSs-ITU treated by ultrasonification. The coprecipitate of INS and ITU was respectively treated by ultrasonification for 0, 5, 10, 20 and 40 min, then enteric-coated with Acryl-Eze. Reported results are means ± SD of 7 mice. Double stars indicate very significant difference (*p* < .01) from other treatments.

ITU-INS binding was studied using microscale thermophoresis (MST). A concentration-dependent binding was found between INS and ITU, and a fully saturated curve with an apparent dissociation constant (*Kd*) of 257 μM was obtained with the INS-ITU polymer (Figure 2(A)). This *Kd* value reflects the relatively weak interactions between the two molecules.

CD was conducted to study the INS structural variation induced by ITU. By Yang’s equation fitting, the CD spectra were recorded (Figure S4, Supplemental File). We set up two conditions: (1) INS was directly mixed with ITU at pH 7.0, and (2) INS was firstly mixed with ITU to form co-precipitate at pH 3.5, then re-dissolved at pH 7.0. As shown in Figure S4 and Table S3 (Supplemental File), ITU could increase α-helix and random coil and reduce β-sheet in INS, no matter directly at pH 7.0 or firstly at pH 3.5 then to pH 7.0. This change makes INS with more compact structure that is favorable for uptake of INS by the intestine.

The coprecipitate of INS and ITU was treated by ultrasonification for different time, then coated with Acryl-Eze to form enteric-coated iMSs-ITU. Without ultrasonification, the average diameter of enteric-coated iMSs-ITU was 2800 nm. After ultrasonification for 5, 10, 20 and 40 min, the average diameter of enteric-coated iMSs-ITU was 1281, 2669,1718 and 1106 nm, respectively (Figure 2(B)). Overall, treatment of coprecipitate by ultrasonification could significantly reduce the diameter of enteric-coated iMSs-ITU. Additionally, the enteric-coated iMSs-ITU were also used for oral administration of diabetic mice. The results showed the hypoglycemic effects of all enteric-coated iMSs-ITU dramatically decreased after treatment by ultrasonification (Figure 2(B)), suggested the special structure of coprecipitate is very important for orally delivering INS. The structure was destroyed by ultrasonification, thereby the oral hypoglycemic effect of iMSs significantly decreased after treatment by ultrasonification.

### Enteric-coated INS delivered by ITU plus SFN (iMSs-ITU-SFN)

**Figure 3.**
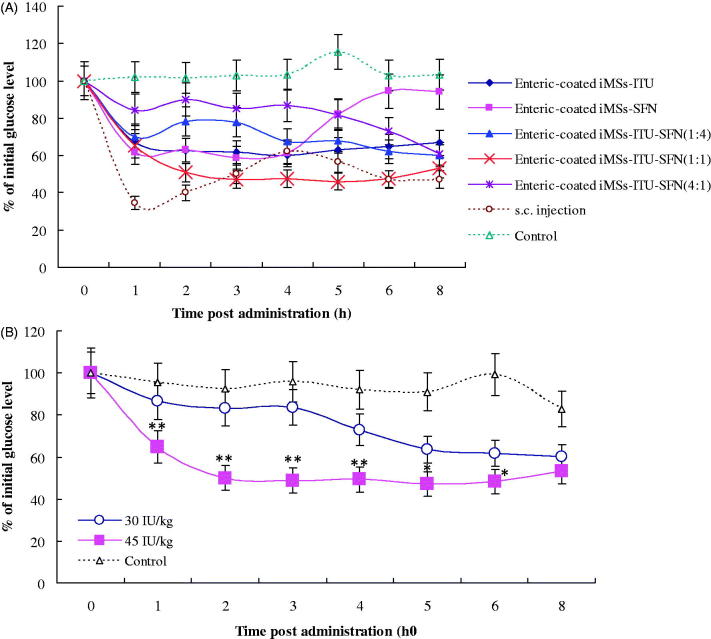
Oral hypoglycemic effect of enteric-coated iMSs-ITU-SFN. (A): Oral hypoglycemic effect of enteric-coated iMSs delivered by ITU plus SFN at different ratios. (B): Oral hypoglycemic effect of enteric-coated iMSs-ITU-SFN (1:1) at different doses. 30 IU/kg and 45 IU/kg: the enteric-coated iMSs-ITU-SFN (1:1) was used for oral administration of diabetic mice at an INS dose of 30 and 45 IU/kg, respectively. Reported results are means ± SD of 7 mice. Double stars and single star indicate very significant difference (*p* < .01) and significant difference (*p* < .05) from other groups, respectively.

Our previously results showed SFN can be used for oral delivery of INS (Zhang et al., 2016). For further improving oral hypoglycemic effect, SFN was added to produce the enteric-coated INS delivered by ITU plus SFN (iMSs-ITU-SFN) at different ratios (ITU: SFN = 1:0, 0:1, 1:1, 4:1, 1:4, respectively, w/w). The results showed the hypoglycemic effect of iMSs-ITU (ITU: SFN = 1:0) was similar to the iMSs-SFN (ITU: SFN = 0:1) in 4 hours postadministration. Thereafter, the blood glucose of former was still sustained at a low level while the latter gradually increased until the end of experiment (Figure 3(A)), suggested the enteric-coated iMSs-ITU has a longer INS release profile with a stronger oral hypoglycemic effect than the enteric-coated iMSs-SFN.

For ITU:SFN = 4:1 (w/w), the iMSs-ITU-SFN showed a slightly oral hypoglycemic effect when compared to control. For ITU:SFN = 1:4, the iMSs-ITU-SFN was stronger for reducing blood glucose level than the above one (ITU:SFN = 4:1), but weaker than the iMSs-ITU or iMSs-SFN (Figure 3(A)). For ITU:SFN = 1:1, the iMSs-ITU-SFN was found with the strongest hypoglycemic effect, which could fast reduce blood glucose to less than 50% of the initial level in 2 h (Figure 3(A)). Compared to *s.c.* injection, the enteric-coated iMSs-ITU-SFN (1:1) showed a sustained INS release profile and a lasted hypoglycemic effect for oral administration (Figure 3(A)).

We further studied the oral dose for enteric-coated iMSs-ITU-SFN (1:1) in diabetic mice. Two INS doses including 45 IU/kg and 30 IU/kg were used, and found both doses could reduce the blood glucose level compared to control, but the high dose of 45 IU/kg was more effective than the low dose of 30 IU/kg (Figure 3(B)).

### Morphology of iMSs

**Figure 4.**
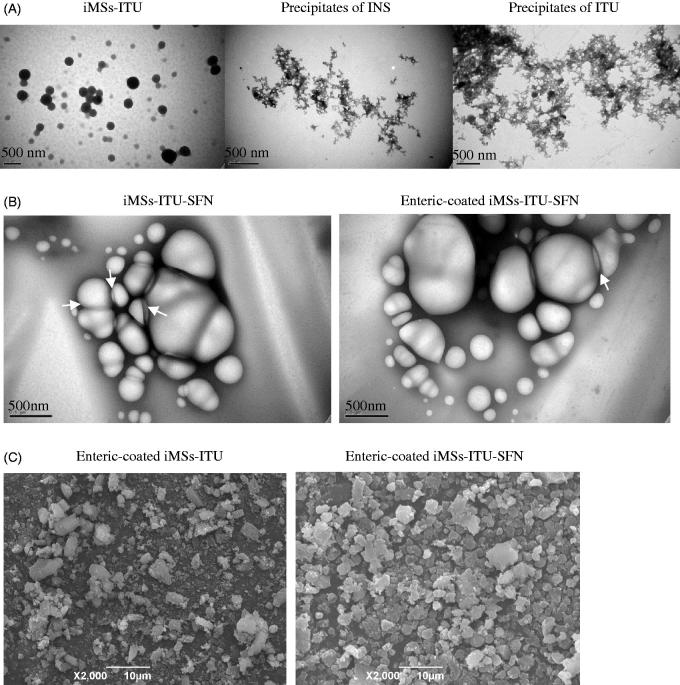
Images of iMSs. (A): The iMSs-ITU without enteric-coating by Acryl-Eze was observed by TEM. The iMSs-ITU without enteric-coating by Acryl-Eze was spherical particles, while the INS alone or ITU alone was irregular polymer. (B): TEM images of iMSs-ITU-SFN (1:1) without (left panel) or with (right panel) enteric-coating by Acryl-Eze. Most of iMSs-ITU-SFN were spherical vesicles, and some vesicles were adhered together (indicated by white arrows). (C): SEM images of enteric-coated iMSs-ITU and iMSs-ITU-SFN. Both of the enteric-coated iMSs-ITU and iMSs-ITU-SFN were spherical microparticles.

Observation by TEM, the iMSs-ITU without enteric-coating was evenly distributed spherical particles, while the INS alone or ITU alone was irregular polymer (Figure 4(A)). At pH 3.5, INS (pI = 5.4) became positively charged while ITU (pI = 2.0) negatively charged, hence forming the co-precipitate of INS and ITU by electrostatic interaction.

The iMSs-ITU-SFN was also observed by TEM. Before enteric-coating by Acryl-Eze, the iMSs-ITU-SFN was spherical vesicles (Figure 4(B)). After coating with Acryl-Eze by lyophilization, the enteric-coated iMSs-ITU-SFN was still spherical vesicles (Figure 4(B)). Notably, some vesicles were adhered together, possibly due to the surfactant activity of ITU and SFN which can promote vesicles adhesion by hydrogen bonds (Arutchelvi et al., 2014).

The surface morphology of enteric-coated iMSs-ITU and iMSs-ITU-SFN was also observed by SEM. The results showed the enteric-coated iMSs-ITU was spherical particles with a size of several micrometers. After addition with SFN, the enteric-coated iMSs-ITU-SFN was still spherical particles with a size similar to the iMSs-ITU (Figure 4(C)).

### Release of enteric-coated iMSs *in vitro*

**Figure 5.**
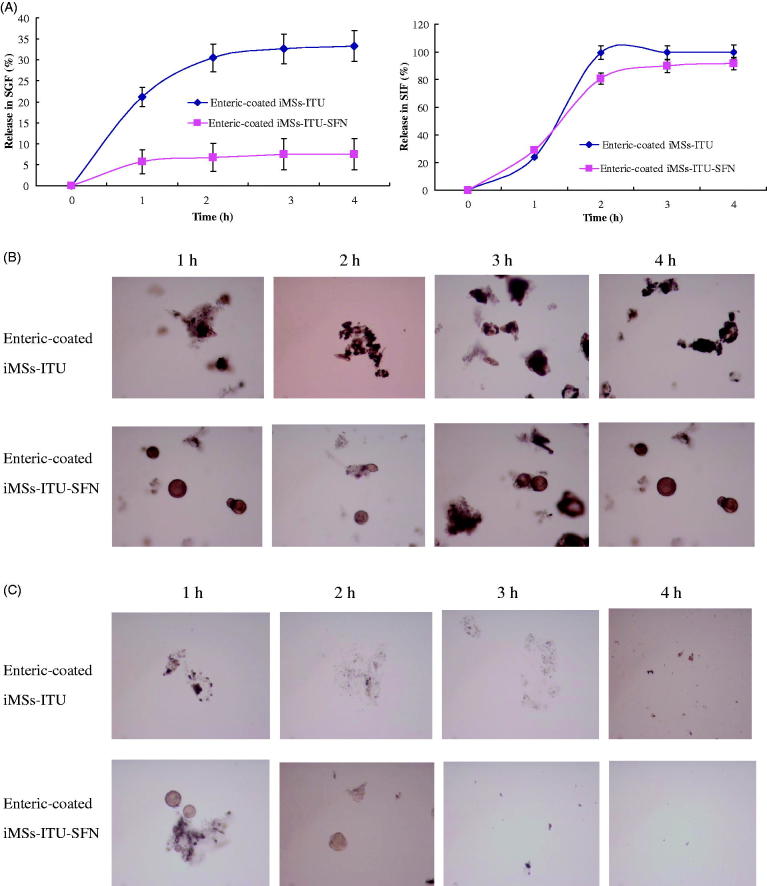
Release of enteric-coated iMSs *in vitro*. (A): Release curves of enteric-coated iMSs in simulated intestinal fluid (SIF) and simulated gastric fluid (SGF). (B): Morphology of enteric-coated iMSs in SGF. (C): Morphology of enteric-coated iMSs in SIF.

As shown in Figure 5(A), only a small amount of INS was released from the enteric-coated iMSs-ITU or iMSs-ITU-SFN after incubation in SGF medium (pH 1.2) for 4 h, with a release rate of 33.1 ± 2.42% and 7.62 ± 0.66%, respectively. This result suggests that the enteric-coated iMSs-ITU-SFN is more stable in the gastric fluid than the enteric-coated iMSs-ITU. While changing the release medium to SIF (pH 6.8), the increase in release rate of INS could be observed both for the enteric-coated iMSs-ITU and iMSs-ITU-SFN. After incubation for 1 h, 24.25 ± 1.73% and 28.12 ± 2.36% of INS was released from the enteric-coated iMSs-ITU and iMSs-ITU-SFN, respectively. After 2 h, almost all INS (98.7 ± 0.13%) was released from the enteric-coated iMSs-ITU, but only 79.04 ± 8.57% released from the enteric-coated iMSs-ITU-SFN. After 4 h, 90.7 ± 8.85% of INS was released from the enteric-coated iMSs-ITU-SFN (Figure 5(A)). These findings suggest that both of the two enteric-coated iMSs can release INS by a sustained profile, but the enteric-coated iMSs-ITU-SFN is stronger for controlled release of INS than the iMSs-ITU.

The morphology of enteric-coated iMSs was observed in the progress of release by a microscopy. After incubation in SGF, the enteric-coated iMSs-ITU tended to cluster together as well the enteric-coated iMSs-ITU-SFN (Figure 5(B)). After incubation in SIF, the enteric-coated iMSs-ITU was gradually collapsed, and this was also observed for the enteric-coated iMSs-ITU-SFN (Figure 5(C)).

### Encapsulation efficiency of INS

Encapsulation efficiency of INS was determined as 65.04% and 66.22% for the enteric-coated iMSs-ITU and iMSs-ITU-SFN respectively, by measuring the INS concentration dissolved in acidic water. This result suggested the addition of SFN has no obvious influence on the encapsulation efficiency of INS in the enteric-coated iMSs.

### Relative bioavailability and OGTT of enteric-coated iMSs

**Figure 6.**
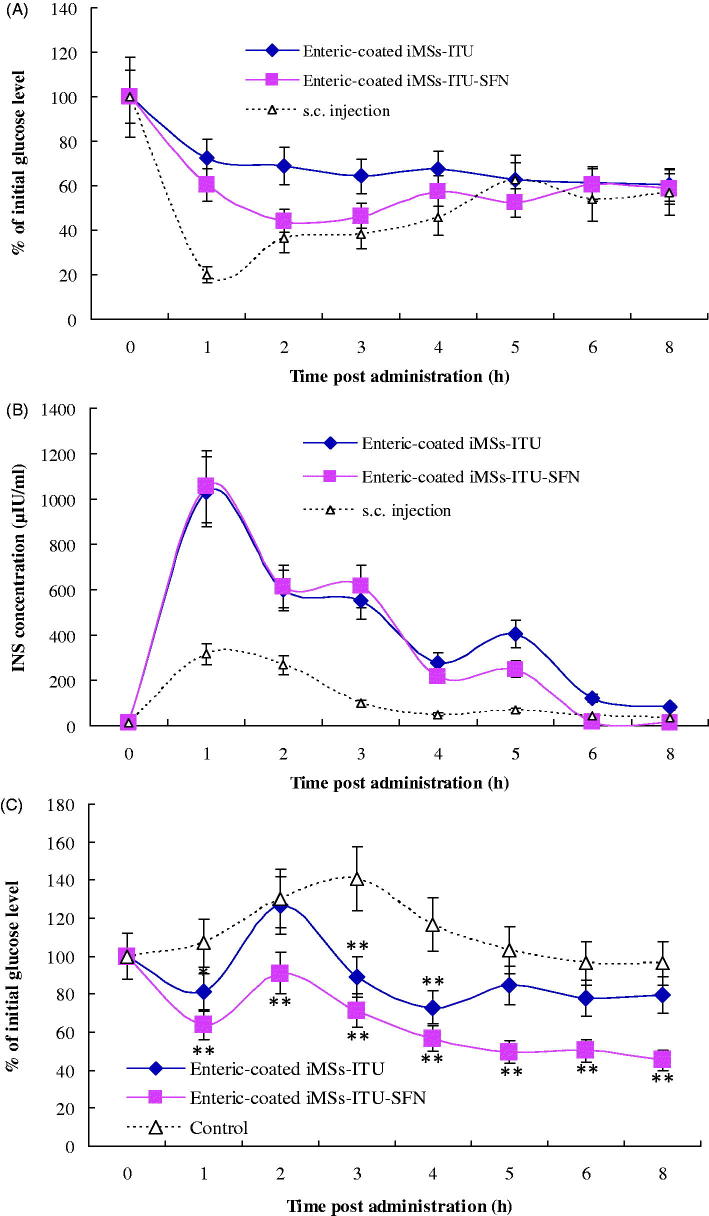
Oral hypoglycemic effect, plasma INS concentration and OGTT of enteric-coated iMSs in diabetes mice. (A): Oral hypoglycemic effect of enteric-coated iMSs in diabetic mice. (B): Plasma INS concentration curves in diabetic mice orally administered with enteric-coated iMSs. (C): OGTT of enteric-coated iMSs in diabetic mice. Reported results are means ± SD of 12 mice. Double stars and single star indicate very significant difference (*p* < .01) and significant difference (*p* < .05) from the control, respectively.

The enteric-coated iMSs-ITU and iMSs-ITU-SFN was used for oral administration of diabetic mice at an INS dose of 45 IU/kg, respectively. As shown in Figure 6(A), both of these two enteric-coated iMSs could reduce the blood glucose level with a controlled release profile after oral administration, but the enteric-coated iMSs-ITU-SFN was stronger for controlling blood glucose than the enteric-coated iMSs-ITU in diabetic mice.

Figure 6(B) is the plasma INS concentrations (μIU/ml) versus time curves. The peak INS concentration of *s.c.* injection was 315 μIU/ml at 1-h postadministration. The peak INS concentration also occurred at 1 h as 1031 μIU/ml and 1054 μIU/ml for the enteric-coated iMSs-ITU and iMSs-ITU-SFN, respectively. The plasma INS concentration of enteric-coated iMSs-ITU-SFN was higher than the enteric-coated iMSs-ITU from 4 to 8 h after administration, indicating the addition of SFN is favorable for the controlled release and uptake of INS by the intestine. The AUC was 2801.08, 3139.73 and 909.73 for iMSs-ITU, iMSs-ITU-SFN and *s.c.* injection, respectively. According to Equation (3), the relative bioavailability of INS was calculated as 6.84% and 7.67% for the enteric-coated iMSs-ITU and iMSs-ITU-SFN, respectively.

OGTT was determined for the enteric-coated iMSs-ITU and iMSs-ITU-SFN, respectively. After oral administration with enteric-coated iMSs, the diabetic mice were orally given with 2 g/kg glucose to mimic a meal. Figure 6(C) shows the comparison of blood glucose in the diabetic mice orally administered with enteric-coated iMSs versus the control. After orally given with glucose, the blood glucose of enteric-coated iMSs increased to the maximal value at 1 h, then gradually decreased to the level significantly lower than the control. Notably, the enteric-coated iMSs-ITU-SFN was much stronger for controlling the postprandial blood glucose level than the iMSs-ITU. The blood glucose decreased to 71.47%, 56.73%, 49.56%, 50.29% and 45.23% of the initial level at 3, 4, 5, 6 and 8 h after oral glucose in the group of enteric-coated iMSs-ITU-SFN. This result suggested the enteric-coated iMSs-ITU-SFN can effectively control the postprandial blood glucose level for a long time in diabetic mice.

## Discussion

To develop oral INS, several barriers should be overtaken such as gastric juice, enzymatic barrier, viscous mucous layer and intestinal epithelium cells (Ismail & Csóka, 2017). For this purpose, numerous strategies are used such as application of protease inhibitors, absorption enhancers, and so on (Kanzarkar et al., 2015; Wong et al., 2016). In this study, the lipopeptides of ITU and SFN were used to orally deliver INS as both of protease inhibitors and absorption enhancers. Lipopeptides, such as SFN and ITU, are the natural products from *Bacillus* species including *B. subtilis*, *B. amyloliquefaciens*, etc., which are widely existed in the *Bacillus*-fermented soybean foods such as Douchi and natto in Eastern Asia (Joshi et al., 2008). Otherwise, a 28-day subacute study also verified that SFN is safety for oral administration at a dose less than 500 mg/kg in rats (Hwang et al., 2009). Thereby, lipopeptides are considered safety for a long oral administration.

Even INS can be accurately released in the intestine by many strategies such as application of biopolymers, some limitations are still needed to be overtaken (Kanzarkar et al., 2015). For example, many biopolymers are easy to be produced on a small laboratory scale, but difficult on a large industrial scale (Lopes et al., 2015; Ma 2015). Comparatively, Acryl-Eze is a widely used commercial enteric-coating polymer system that can accurately release INS in the intestine. A good delivery system should contain biologically active INS upon the encapsulation process. Accordingly, lyophilization was used to produce the enteric-coated iMSs with a slight influence on the INS activity in this study. The amount of Acryl-Eze significantly influenced the oral hypoglycemic effect of enteric-coated iMSs by determining the thickness of enteric-coating layer. Here, we found a small amount of Acryl-Eze could not form an effective enteric-coating film, and a large amount of Acryl-Eze formed an excessive enteric-coating film, both were not favorable for efficiently delivering INS in the intestine. Only an appropriate amount of Acryl-Eze could form a moderate enteric-coating film for accurately delivering INS in the intestine.

The enteric-coated iMSs were spherical particles with an average size about 3 µm. Absorption of particles in the GI tract occurs through various sites depending upon their sizes (Sharma et al., 2015). After oral administration, the enteric-coated iMSs (∼ 3 µm) might be absorbed by the phagocytosis of intestinal macrophages (Wong et al., 2016). In addition to direct absorption, most of enteric-coated iMSs were collapsed in the intestine, then the INS was released following absorbed via simple diffusion by the promotion of ITU or ITU plus SFN. Both SFN (Zhang et al., 2016) and ITU could bind with and promote INS across the intestinal epithelium. Compared to iMSs-ITU, INS released from the enteric-coated iMSs-ITU-SFN was easier to be absorbed by the intestine. As a result, the enteric-coated iMSs-ITU-SFN showed a stronger oral hypoglycemic effect than the iMSs-ITU. The oral bioavailability was also superior for the enteric-coated iMSs-ITU-SFN (7.67%) than the iMSs-ITU (6.84%).

Just as SFN, ITU could weakly bind with INS (*Kd* = 257 μM). Lipopeptides firstly bind to INS by electrostatic interactions, then further interact each other by hydrophobic interactions (Zhang et al., 2016, 2017). Here, ITU was more effective for orally delivering INS than SFN. This might be due to the difference of these two lipopeptides. SFN can induce INS structure more flexible by its strong hydrophobility (Zhang et al., 2016, 2017). Flexible INS is easier to penetrate across the intestinal epithelium, but the INS activity might be weakened by the excessive flexible structure such as β-sheet in the protein. However, ITU is more hydrophilic than SFN, which tends to induce INS structure more compact by its hydrophilicity. Generally, the activity of protein is determined by its rigid structure such as α-helix, and hence, the INS activity is less influenced by ITU compared to SFN. As a result, the enteric-coated iMSs-ITU showed a stronger hypoglycemic effect than the iMSs-SFN. Moreover, ITU plus SFN (1:1) was more effective for orally delivering INS, possibly due to it can better bind with and promote INS across the intestine than ITU, SFN, or ITU plus SFN at other ratios (1:4 or 4:1).

Oral INS can mimic the natural physiologic route of INS through the portal vein and direct engagement of the liver (Akbari et al., 2016). Owing to the first-pass hepatic extraction, 50% of the INS is degraded in the liver (Kanzarkar et al., 2015). Overall, INS level is significantly reduced in the systemic circulation after oral administration. However, for *s.c.* injection, most of INS exists in the systemic circulation, and only a small amount (approximately 20%) reaches the liver with the first-pass hepatic extraction (Fonte et al., 2015). Accordingly, oral INS is often found with a poor relative bioavailability compared to *s.c.* injection due to the first-pass hepatic extraction (Kanzarkar et al., 2015). Here, we used blood samples from the tail vein to calculate the oral INS bioavailability. Compared to *s.c.* injection, the relative bioavailability was 6.84% and 7.67% for the enteric-coated iMSs-ITU and iMSs-ITU-SFN, respectively.

We previously reported that the oral INS delivered by SFN has a relative bioavailability of 12.48% in diabetic mice (Zhang et al., 2016). In fact, this value was achieved by comparison with the *s.c.* INS dose of 2 IU/kg, but the relative bioavailability was achieved by compared to the *s.c.* INS dose of 1 IU/kg in this study. Moreover, the oral hypoglycemic effect of 45 IU/kg INS in this study was much stronger than the previously reported 90 IU/kg. All of these data indicate that the relative bioavailability is influenced by many factors such as the INS dose for oral and *s.c.* administration, body sites for blood collection as well as animal species used. For example, the oral INS dose for mice is generally about twofold for rats that will cause a lower relative bioavailability in mice compared to rats. Also, INS from the hepatic portal vein dose not experience the first-pass hepatic extraction, hence showing a higher oral bioavailability than the INS from systemic circulation such as tail vein in this study. Here, even the bioavailability was not very high due to the first-pass hepatic extraction, the enteric-coated iMSs was effective on controlling the postprandial blood glucose in diabetic mice similar to *s.c.* injection.

In this study, the enteric-coated iMSs-ITU and iMSs-ITU-SFN showed an encapsulation efficiency of 65.04% and 66.22%, respectively, indicating the addition with SFN can not obviously improve the encapsulation efficiency of INS. Thereby, more studies are necessary to improve the INS encapsulation efficiency for a stronger oral hypoglycemic effect and higher oral bioavailability.

## Conclusions

Collectively, lipopeptides are very potential for the oral delivery of INS or other protein drugs due to their nontoxic, biodegradable and absorption-enhancing properties. INS was protected by the enteric-coating with Acryl-Eze in the stomach, following absorbed in the intestine by the promotion of lipopeptides. As a result, the enteric-coated iMSs delivered by lipopeptides showed a prolonged hypoglycemic effect after oral administration, indicating they are very potential for the every day control of blood glucose in the face of continuous influx of glucose and possibly other carbohydrates in diabetic patients.

## Supplementary Material

IDRD_Qi_et_al_Supplemental_Content.doc
