# Supplementary material for: Enteric-coated insulin microparticles delivered by lipopeptides of iturin and surfactin
Source: Drug Deliv. 2017 Dec 10;25(1):23–34. doi: 10.1080/10717544.2017.1413443 (PMC6058518; doi:10.1080/10717544.2017.1413443)
Supplement: IDRD_Qi_et_al_Supplemental_Content.doc [file IDRD_A_1413443_SM2009.doc]

**Supplementary data**

**Figure S1**

(a)

CH_3_(CH_2_)_10_CHCH_2_CO—L-Glu—L-Leu—D-Leu—L-Val—L-Asp—D-Leu—L-Leu

O

(b)

C_11-13_CHCH_2_CO—L-Asn—D-Tyr—D-Asn—L-Gln—L-Pro—D-Asn—L-Ser

HN

**Fig. S1** **Schematic structures of lipopeptides.** (a): surfactin (SFN); (b): iturin (ITU).

**Figure S2**

(a) (b)

P_1.1_


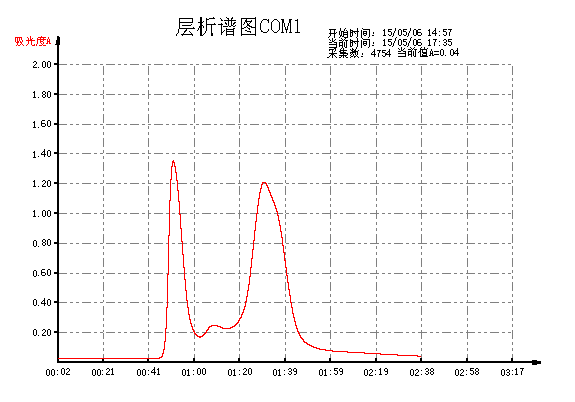

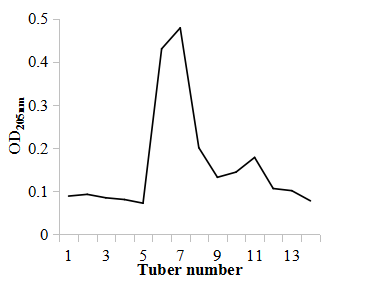


OD_205nm_

P_1_

P_2_

Elution time

P_1.2_

(c)


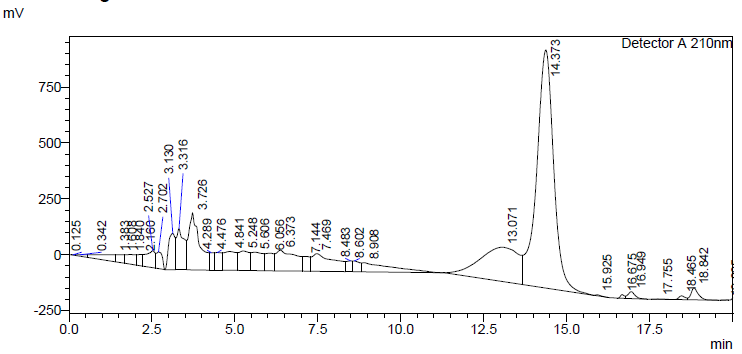


Iturin

(d)


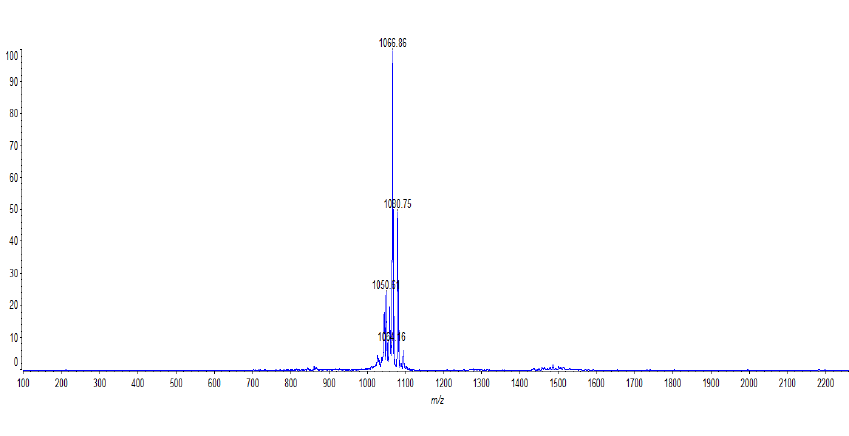


**Fig. S2 Purification and characterization of ITU.** (a): Purification of ITU from the n-butanol extracted components by Sephadex G25. (b): Purification of ITU by silica gel column. (c): Analysis of ITU by RP-HPLC. (d): Analysis of ITU by MALDI-TOF.

**Figure S3**


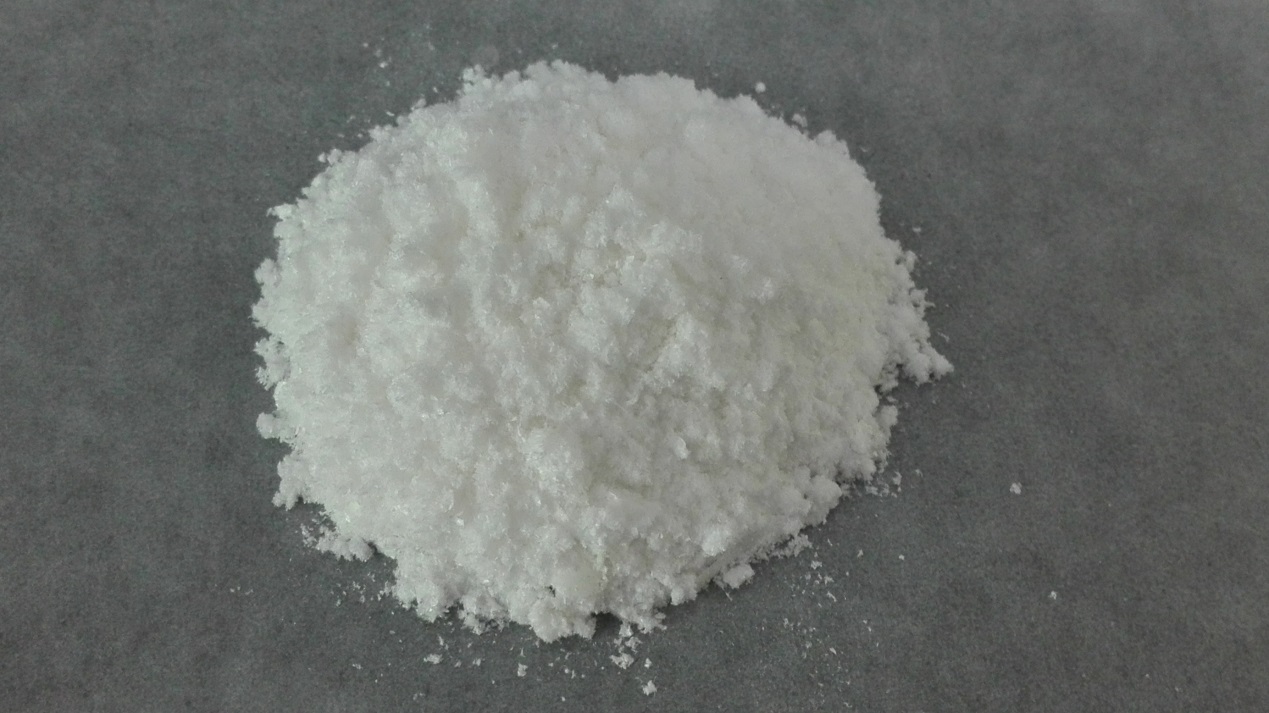


**Fig. S3 Morphology of enteric-coated iMSs-ITU.**

**Figure S4**

INS Only

1:0.5

1 : 1

1 : 2

1 : 3


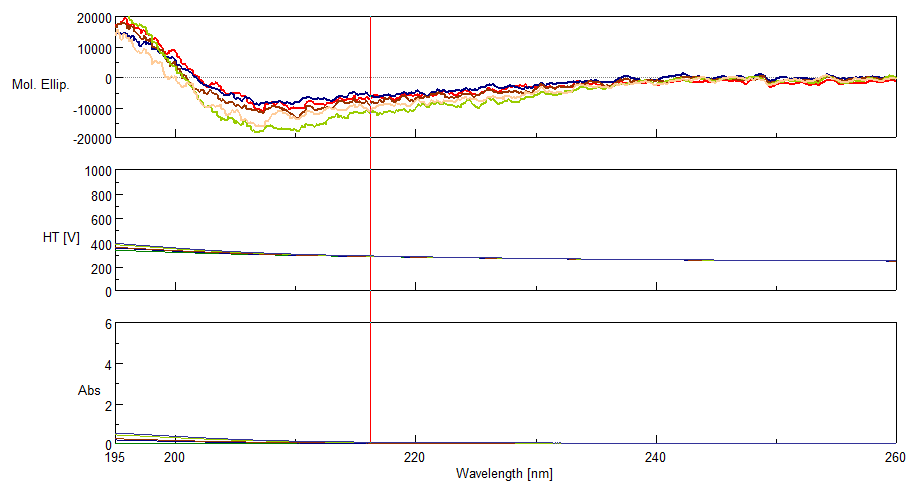

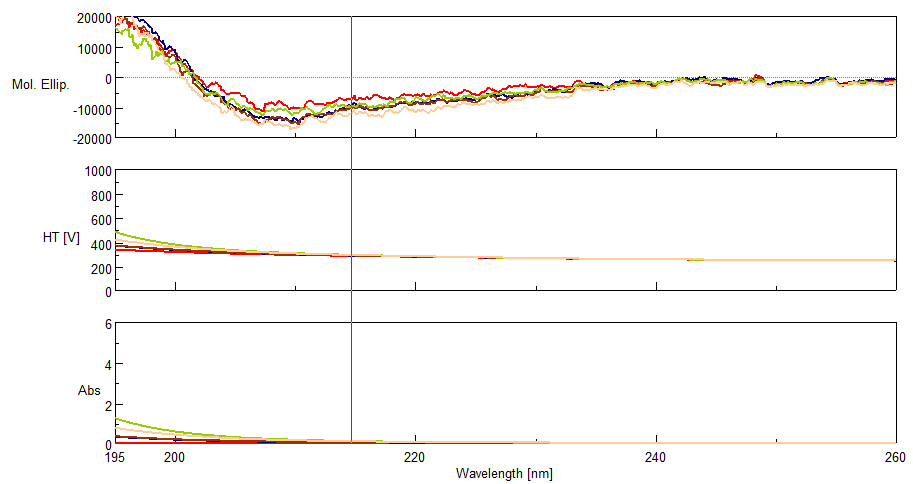


pH=7.0

INS Only

1:0.5

1 : 1

1 : 2

1 : 3


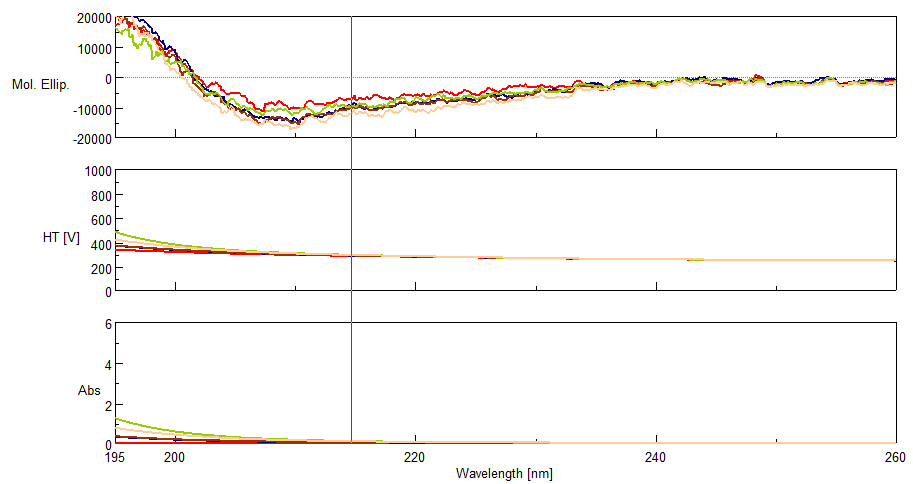

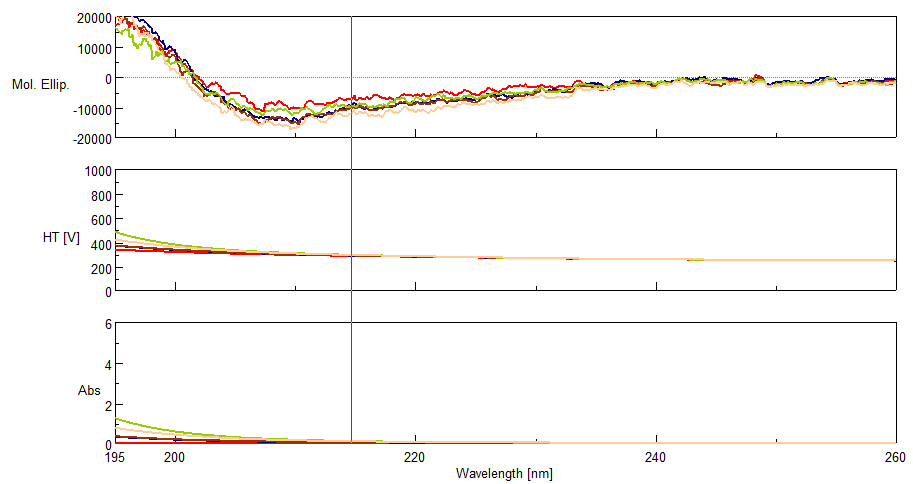


pH=3.5 7.0

(C)

**Fig. S4** **Circular diehroism spectroscopy of INS influenced by ITU.** 1:0.5, 1:1, 1:2 and 1:3 means the ratio of INS to ITU (m/m).

Table S1 Preparation of mixtures containing different amounts of INS and ITU.

| INS:ITU (w/w) | INS (mg/ml) | ITU (mg/ml) |
| --- | --- | --- |
| INS only | 0.025 | 0 |
| ITU only | 0 | 0.025 |
| 1:0.5 | 0.025 | 0.0125 |
| 1:1 | 0.025 | 0.0250 |
| 1:2 | 0.025 | 0.050 |
| 1:3 | 0.025 | 0.075 |

Table S2 Preparation of enteric-coated iMSs delivered by ITU plus SFN at different ratios.

| ITU:SFN (w/w) | ITU | SFN |
| --- | --- | --- |
| ITU only | 1.0 mg | 0 |
| SFN only | 0 | 1.0 mg |
| 1:1 | 0.5 mg | 0.5 mg |
| 4:1 | 0.8 mg | 0.2 mg |
| 1:4 | 0.2 mg | 0.8 mg |

Table S3 The secondary structure of INS influenced by ITU

|  | INS:ITU (w/w) | Helix% | Beta% | Turn% | Random% |
| --- | --- | --- | --- | --- | --- |
| INS only |  | 22.4 | 63.4 | 3.2 | 11.0 |
| pH=7.0 | 1:0.5 | 16.1 | 69.6 | 0.8 | 13.5 |
|  | 1:1 | 22.2 | 60.1 | 0 | 17.8 |
|  | 1:2 | 35.0 | 38.9 | 0 | 26.2 |
|  | 1:3 | 23.8 | 45.8 | 0 | 30.4 |
| pH=3.5-7.0 | 1:0.5 | 31.4 | 55.4 | 0 | 13.2 |
|  | 1:1 | 30.0 | 50.7 | 0 | 19.3 |
|  | 1:2 | 25.9 | 47.9 | 0 | 26.1 |
|  | 1:3 | 33.8 | 39.0 | 0 | 27.2 |
